# Reconsidering early RRT in leptospirosis AKI: a question of timing criteria

**DOI:** 10.1186/s13613-025-01544-x

**Published:** 2025-09-02

**Authors:** Pei-Chen Wu, Thomas Tao-Min Huang

**Affiliations:** 1 Division of Nephrology, Department of Internal Medicine, MacKay Memorial Hospital, Taipei City, Taiwan; 2 Department of Medicine, MacKay Medical College, New Taipei City, Taiwan; 3 Taiwan Society of Emergency and Critical Care Medicine, Taipei City, Taiwan; 4 Department of Internal Medicine (Nephrology and Critical Care), National Taiwan University Hospital and College of Medicine, No. 7, Chung Shan S. Rd., Zhong-Zheng District, Taipei City, 10008 Taiwan

Dear Editor-in-Chief,

We read with great interest the recent article by Julien et al. investigating the timing of renal replacement therapy (RRT) in patients with leptospirosis-associated acute kidney injury, AKI-L) [1]. The authors should be commended for addressing this clinically relevant question through an emulated target trial design, one that is especially valuable given the logistical constraints of conducting randomized controlled trials in this context.

However, we propose an alternative interpretation of the findings: the observed adverse outcomes in the early RRT group may not be inherent to early dialysis initiation per se, but rather reflect a fundamental limitation in using KDIGO Stage 3 as a surrogate trigger for RRT initiation in leptospirosis.

The authors define “early RRT” as initiation within 48 h of hospital admission among patients with KDIGO Stage 3 AKI. Yet AKI-L exhibits distinct pathophysiological and clinical profiles, including preserved urine output, hypokalemia, and tubular sodium loss—factors which typically delay the onset of conventional RRT indications. Indeed, in the manuscript, the authors note that 64% of patients in the early RRT group did not exhibit hyperkalemia, BUN > 30 mmol/L, or severe metabolic acidosis at the time of RRT initiation. This suggests a sizeable portion of RRTs may have been initiated preemptively based on creatinine rise alone [1].

The increased incidence of new-onset or worsening CKD in the early group (OR 2.74, *p* = 0.012) raises the possibility that premature initiation of RRT—when not clinically indicated—may contribute to downstream harm, perhaps via hemodynamic instability or sustained RRT-dependence [1]. Rather than undermining early RRT altogether, these findings argue against the validity of applying KDIGO Stage 3 as a one-size-fits-all threshold in AKI-L.

This interpretation is supported by major randomized controlled trials in broader AKI populations, such as AKIKI [2], IDEAL-ICU [3], STARRT-AKI [4], and ELAIN [5]. While the ELAIN trial did show a benefit of early RRT initiation in a highly selected population using a biomarker-guided strategy, it also emphasized the importance of individualized patient selection and timely initiation guided by both functional and biochemical markers [5]. Together, these studies underscore that early RRT may be beneficial only when guided by robust, patient-specific criteria—not staging alone [2–4].

Julien et al.‘s own findings further strengthen this argument. Of the patients in the early RRT group, a majority did not have any classical dialysis indications at the time of RRT initiation. Additionally, the delayed group revealed that 75% of patients ultimately avoided RRT altogether and yet achieved favorable renal outcomes, indicating that Stage 3 AKI alone may not reliably identify patients who truly require RRT [1]. Furthermore, AKI-L often progresses in a biphasic manner and may be reversible with supportive therapy, suggesting that relying on staging thresholds such as KDIGO Stage 3 risks unnecessary initiation of RRT, with potential long-term iatrogenic harm (e.g., HIRRT and dialysis dependency).

In conclusion, this study reinforces the need to move beyond creatinine-centric definitions of “early” RRT in leptospirosis. We advocate for research efforts that refine patient selection criteria, with particular attention to the unique trajectory of AKI-L. Such efforts may better identify those truly likely to benefit from RRT while sparing others from unnecessary intervention.

## Data Availability

Not applicable.

## Abbreviations

AKI: Acute kidney injury
AKI-L: Leptospirosis-associated acute kidney injury
CKD: Chronic kidney disease
KDIGO: Kidney Disease Improving Global Outcomes
RRT: Renal replacement therapy
HIRRT: Hemodynamic instability-related renal replacement therapy
